# Epidemiological and Metabolic Characteristics of *Clostridium perfringens* Type B Isolates from Dairy Goat Kids with Diarrhea in Inner Mongolia

**DOI:** 10.3390/ani16132041

**Published:** 2026-07-02

**Authors:** Ziqi Meng, Lei Yang, Fan Bai, Ziqiang Zhang, Shaoyin Fu, Na Chen, Yu Guo, Nicolas Lopez-Villalobos, Qinan Zhao

**Affiliations:** 1 Inner Mongolia Academy of Agricultural & Animal Husbandry Sciences, No. 22 Zhaojun Road, Hohhot 010031, China; 18047131906@163.com (Z.M.); yanglei0602@126.com (L.Y.); shmilycccp@126.com (F.B.); hnzhangziqiang@163.com (Z.Z.); fushao1234@126.com (S.F.); 13654844851@163.com (N.C.); nmgnkyguoyu@163.com (Y.G.); 2 School of Agriculture and Environment, Massey University, Palmerston North 4442, New Zealand; n.lopez-villalobos@massey.ac.nz

**Keywords:** *Clostridium perfringens*, dairy goat, kids, diarrhea, toxin genotyping, antimicrobial resistance, metabolomics

## Abstract

Diarrhea in neonatal dairy goat kids is a major disease affecting the dairy goat industry, yet the predominant bacterial pathogens in Inner Mongolia remain unclear. This study investigated the main bacterial pathogens and characterized the toxin genotypes, antimicrobial resistance, and metabolic features of *Clostridium perfringens*. A total of 1169 fecal samples from diarrheic kids (<15 days old) were analyzed. *C. perfringens* showed the highest positive rate (56.06%), followed by *Escherichia coli* (46.71%) and *Salmonella* spp. (24.54%), and was positively correlated with bloody diarrhea (r = 0.31). Among 73 isolates, type B (67.1%) was predominant. Most isolates were highly susceptible to tetracycline, cefoxitin, penicillin, and metronidazole, while resistance to ampicillin was relatively high. Metabolomic analysis revealed that type B strains exhibited enhanced metabolic activity compared with type C strains. In conclusion, type B *C. perfringens* is the predominant pathogen and shows metabolic advantages, providing a basis for targeted control strategies.

## Simple Summary

Diarrhea in kids is a common disease in dairy goat farming and can cause significant economic losses. This study investigated the main bacterial causes of diarrhea in kids in Inner Mongolia by analyzing 1169 fecal samples from affected animals. The results showed that *Clostridium perfringens* was the most common pathogen, followed by *Escherichia coli* and *Salmonella*. Among the *Clostridium perfringens* strains, type B was the dominant type. Most isolates were sensitive to commonly used antibiotics, although resistance to ampicillin was relatively high. Further analysis showed that type B strains had more active metabolism, which may help them grow faster and cause more severe disease. These findings improve our understanding of diarrhea in dairy goat kids and provide useful information for disease prevention and treatment.

## 1. Introduction

Dairy goat production plays an increasingly important role in the agricultural economy of many regions, providing high-quality milk and dairy products for human consumption [1]. In China, the dairy goat industry has expanded rapidly in recent decades, particularly in Inner Mongolia. The increasing intensification of production systems has brought substantial economic benefits but has also introduced new challenges. As veterinarians and farmers focus more on improving productivity, the incidence of health problems such as neonatal diarrhea, mastitis, and foot diseases has risen concurrently [2]. These conditions not only compromise animal welfare but also lead to significant economic losses through increased mortality, reduced growth rates, and treatment costs.

Neonatal diarrhea is one of the most common and economically devastating diseases affecting lambs and goat kids worldwide [3]. A wide range of etiological factors contribute to the development of diarrhea in young ruminants. Bacterial pathogens including *Escherichia coli*, Salmonella spp., and *Clostridium perfringens* are frequently implicated, but viral agents like rotavirus and coronavirus, parasitic infections such as coccidia and Cryptosporidium, nutritional imbalances including improper colostrum intake or abrupt feed changes, and poor management practices such as inadequate hygiene, overcrowding, and stressful weaning also play important roles [4,5,6]. In many cases, multiple factors act synergistically, making diagnosis and control particularly challenging.

Among bacterial pathogens, *Clostridium perfringens* is a major opportunistic pathogen that causes enterotoxemia and hemorrhagic enteritis in lambs and goat kids [7]. Its pathogenicity mainly depends on the production of toxins, which also forms the basis for its classification into seven toxinotypes (A–G) [8]. Notably, the distribution of toxinotypes varies across different geographic regions, and the predominant genotype causing diarrhea in dairy goat kids in Inner Mongolia remains unclear. However, the metabolic mechanisms underlying the predominance of specific *C. perfringens* toxinotypes have not been systematically investigated. Beyond toxin-mediated virulence, accumulating evidence highlights the importance of metabolic adaptation in bacterial fitness and ecological advantage [9].

To address this gap, this study aimed to identify the major bacterial pathogens responsible for diarrhea in dairy goat kids in Inner Mongolia through epidemiological investigation. Subsequently, toxinotyping, antimicrobial resistance testing, and in vitro bacteriostatic activity analysis of traditional Chinese medicines against the dominant pathogen *C. perfringens* were performed. Additionally, untargeted metabolomics was employed to compare metabolic differences among predominant toxinotypes, thereby providing systematic data support and a theoretical basis for the scientific control of this disease.

## 2. Materials and Methods

### 2.1. Samples

**Figure 1:**
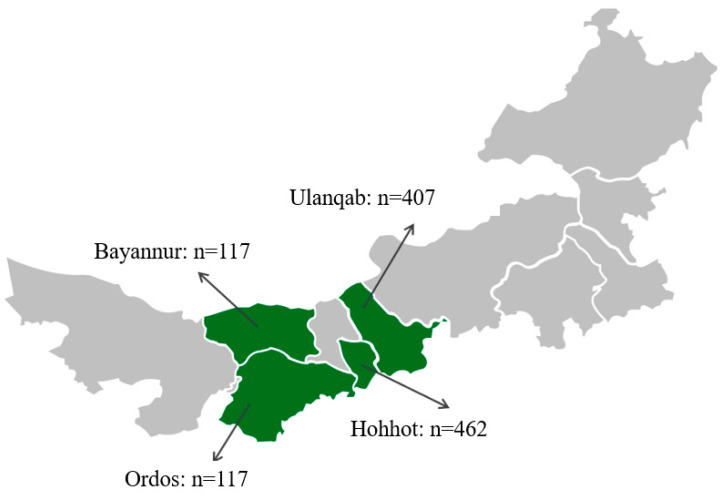
Distribution of 1169 fecal sample collections from dairy goat kids with diarrhea in the Inner Mongolia Autonomous Region.

This study was conducted from February to June 2022 in large-scale dairy goat farms located in Hohhot, Ulanqab, Ordos, and Bayannur of the Inner Mongolia Autonomous Region, China (Figure 1). The goat kids were a crossbreed of Saanen and Guanzhong dairy goats and were artificially fed with milk replacer. A total of 1169 fecal samples were collected from diarrheic dairy goat kids for etiological analysis. All sampled kids were within 15 days of age and exhibited clinical signs including lethargy, abdominal pain, and diarrhea. The diarrheic feces were watery or mucoid, yellowish-green or grayish-yellow in color, and some were blood-tinged. No affected animal had received any medication before sampling. Rectal swabs were taken, stored at 4 °C, and transported to the laboratory within 12 h. Blood was collected from the jugular vein, and sera were separated and stored at −80 °C.

### 2.2. Bacterial Isolation and Identification

Each swab head was cut into a sterile centrifuge tube containing 1 mL sterile physiological saline, vortexed for 1 min to form a suspension, and serially diluted. A sterile loop was used to streak the diluted suspension onto blood agar plates (Qingdao Hope Bio-Technology Co., Ltd., Qingdao, China) by the quadrant streak method, and the plates were incubated at 37 °C for 24 h under both aerobic and anaerobic conditions. Colony morphology and hemolytic characteristics were observed, and single suspected colonies showing β-hemolysis or double-zone hemolysis were selected and subcultured on the following media for preliminary identification: nutrient agar (Qingdao Hope Bio-Technology Co., Ltd., Qingdao, China) to observe basic colony morphology and pigment production and to screen for abnormal bacteria; MacConkey agar (Qingdao Hope Bio-Technology Co., Ltd., Qingdao, China) for selective isolation of *Escherichia coli* and *Salmonella* spp.; and TSC (tryptose sulfite cycloserine) agar (Qingdao Hope Bio-Technology Co., Ltd., Qingdao, China) under anaerobic conditions for initial isolation and identification of *Clostridium perfringens*.

### 2.3. Isolation and Culture of Pathogenic Bacteria

*Salmonella* spp. and *Escherichia coli* were identified using a commercial EasyID biochemical identification kit (Guangdong Huankai Microbial Technology Co., Ltd., Guangzhou, China) following the manufacturer’s instructions. *Salmonella* identification included TSI, KCN, lysine decarboxylase, indole, carbohydrate fermentation (mannitol, sorbitol, dulcitol, salicin), ONPG, malonate, urease, and H_2_S tests. *E. coli* was confirmed by IMViC tests (indole, methyl red, Voges–Proskauer, citrate). *Clostridium perfringens* was identified according to DB15/T 3438-2024 [10] by anaerobic enrichment in FTG medium, followed by the iron milk test at 46 °C for stormy fermentation and LS medium culture to observe gas production and medium blackening.

### 2.4. Gram Staining

A pure colony was smeared, stained sequentially with crystal violet for 1 min and Gram’s iodine for 1 min, decolorized for 30 s, counterstained with safranin for 1 min, air-dried, and observed under a light microscope.

### 2.5. Serum ELISA Antibody Detection

Sera was thawed at room temperature. Antibodies against *E. coli*, *Salmonella* spp., and *C. perfringens* were detected using commercial ELISA kits (Shanghai Moshaike Bio-Tech Co., Ltd., Shanghai, China), strictly following the manufacturer’s instructions.

### 2.6. Toxin Gene Detection of Clostridium perfringens

Genomic DNA of *Clostridium perfringens* was extracted using a commercial kit (Tiangen Biotech Co., Ltd., Beijing, China). The toxin genes cpa, cpb, and etx were detected by PCR using specific primers (Sangon Biotech Co., Ltd., Shanghai, China). The primers were designed based on previously reported sequences [11,12]. Amplification was performed under standard conditions using a T100 Thermal Cycler (Bio-Rad Laboratories, Inc., Hercules, CA, USA), and PCR products were analyzed by 1% agarose gel electrophoresis using a GelDoc XR+ Gel Documentation System (Bio-Rad Laboratories, Inc., Hercules, CA, USA). CVCC53 (China Veterinary Culture Collection Center, Beijing, China) and sterile double-distilled water were used as positive and negative controls, respectively. The primers used for toxin gene detection are listed in Table 1.

### 2.7. Antimicrobial Susceptibility Testing

The minimum inhibitory concentrations of penicillin, metronidazole, clindamycin, ampicillin, cefoxitin, and tetracycline (all from Sigma-Aldrich Corp., St. Louis, MO, USA) against *Clostridium perfringens* were determined using the broth microdilution method according to CLSI guidelines (CLSI M100, Clinical and Laboratory Standards Institute, Wayne, PA, USA). Bacterial suspensions were prepared from single colonies and adjusted to approximately 1 × 10^6^ CFU/mL In cation-adjusted Mueller-Hinton broth (CAMHB; Qingdao Hope Bio-Technology Co., Ltd., Qingdao, China)Serial twofold dilutions of antibiotics were prepared in 96-well plates, followed by inoculation and anaerobic incubation at 37 °C for 24 h. The minimum inhibitory concentrations values were interpreted as susceptible, intermediate, or resistant based on CLSI breakpoints. The interpretive criteria for antimicrobial susceptibility testing based on CLSI guidelines are shown in Table 2.

### 2.8. Untargeted Metabolomics Analysis

A total of six type B and six type C *Clostridium perfringens* samples (culture supernatants and cell pellets) were collected for metabolomic analysis. Metabolites were extracted using pre-chilled methanol/acetonitrile/water (2:2:1, *v*/*v*/*v*; all from Merck KGaA, Darmstadt, Germany), followed by vortexing, sonication, centrifugation, and vacuum drying. The dried extracts were reconstituted in acetonitrile/water (1:1, *v*/*v*; Merck KGaA, Darmstadt, Germany) prior to LC-MS analysis. LC-MS analysis was performed by Applied Protein Technology (Shanghai, China) using a liquid chromatography-tandem mass spectrometry system, and metabolite identification and quantification were conducted based on retention time, accurate mass (error < 10 ppm), and MS/MS spectra against their in-house database.

### 2.9. Data Analysis

Data were organized using Microsoft Excel 2021 (Microsoft Corp., Redmond, WA, USA). Pearson correlation analysis was performed to evaluate the association between clinical manifestations and pathogen detection results, with *p* < 0.05 considered statistically significant. Metabolomic data were processed using XCMS v3.14.0 (Scripps Research, La Jolla, CA, USA) for peak detection and retention time alignment. Differential metabolites were screened based on variable importance in projection (VIP) > 1, *p* < 0.05, and fold change (FC) > 1.5 or < 0.67. Quality control was assessed by total ion chromatogram overlap, correlation of QC samples (|r| > 0.9), and the proportion of peaks with relative standard deviation (RSD) ≤ 30% exceeding 80%.

### 2.10. Isolation and Biochemical Identification of Major Bacterial Pathogens

**Figure 2:**
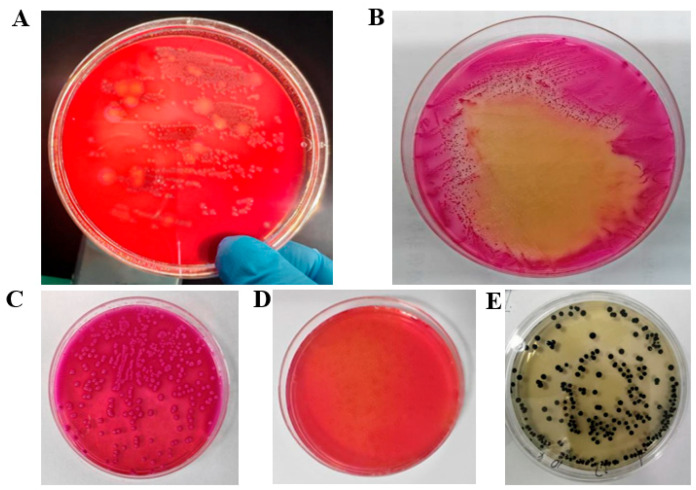
Colony morphology of major bacterial pathogens isolated from dairy goat kids with diarrhea. (**A**) Hemolytic colonies on blood agar; (**B**) mixed growth of *Escherichia coli* and *Salmonella* spp. on MacConkey agar; (**C**) typical colonies of *E. coli* on MacConkey agar; (**D**) typical colonies of *Salmonella* spp. on MacConkey agar; (**E**) black colonies of *Clostridium perfringens* on TSC agar.

A total of 1169 fecal samples from diarrheic kids were subjected to bacterial isolation. Hemolytic zones were observed on blood agar plates (Figure 2A). After subculture on MacConkey agar, two distinct colony types were identified, including pink colonies consistent with *Escherichia coli* and colorless to pale colonies consistent with *Salmonella* spp. (Figure 2B). Purified *E. coli* colonies appeared pink, medium-sized, and smooth (Figure 2C), whereas *Salmonella* colonies were smaller, smooth, and pale to slightly yellowish (Figure 2D). In addition, black colonies formed on TSC agar under anaerobic conditions (Figure 2E), indicating the presence of *Clostridium perfringens*.

**Figure 3:**
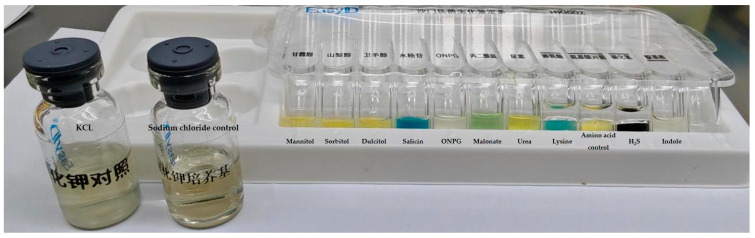
Chromogenic results of biochemical identification of *Salmonella* spp.

**Figure 4:**
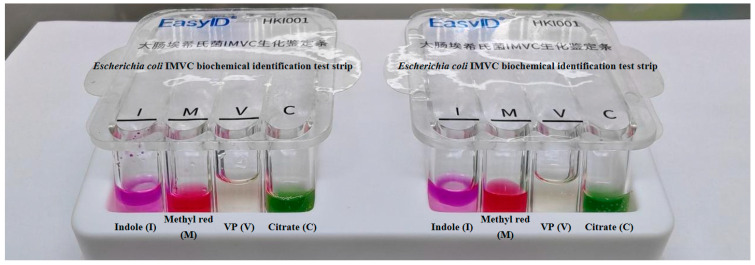
Chromogenic results of biochemical identification of *Escherichia coli*.

**Figure 5:**
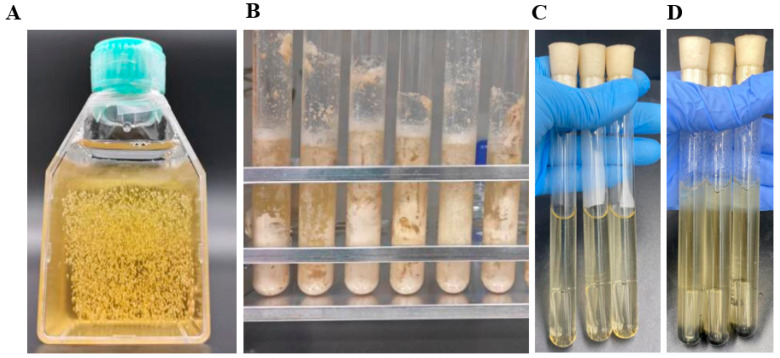
Biochemical identification of *Clostridium perfringens*. (**A**) Growth in FTG medium; (**B**) stormy fermentation in iron milk medium; (**C**) gas production in LS medium; (**D**) black precipitate formation in LS medium.

Biochemical identification showed that the isolates were consistent with the characteristics of *Salmonella* spp., *Escherichia coli*, and *Clostridium perfringens* according to standard criteria. The chromogenic and biochemical reaction profiles of *Salmonella* spp. and *E. coli* are presented in Figure 3 and Figure 4, respectively, with detailed results summarized in Table 3 and Table 4. The isolates of *C. perfringens* exhibited typical features, including stormy fermentation in iron milk medium and gas production with blackening in LS medium (Figure 5).

**Figure 6:**
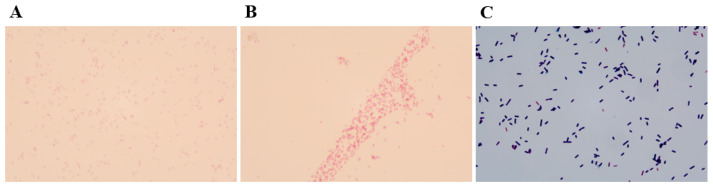
Gram staining of bacterial isolates. (**A**) *E. coli*; (**B**) *Salmonella* spp.; (**C**) *C. perfringens*.

Gram staining further confirmed the bacterial morphology: *E. coli* and *Salmonella* spp. appeared as Gram-negative short rods, whereas *C. perfringens* showed Gram-positive large rods (Figure 6).

Serological analysis by ELISA revealed that, in the diarrheic kid group (*n* = 974), the highest antibody-positive rate was observed for *C. perfringens* (56.06%, 546/974), followed by *E. coli* (46.71%, 455/974) and *Salmonella* spp. (24.54%, 239/974). In contrast, the healthy control group (*n* = 157) showed markedly lower positive rates of 1.27%, 8.28%, and 3.82%, respectively (Table 5).

## 3. Results

### 3.1. Clinical Manifestations of Diarrheic Kids

During sample collection, diarrheic kids were predominantly younger than 14 days of age and commonly exhibited depression, reduced appetite, and varying degrees of dehydration. Based on fecal characteristics, the clinical manifestations could be categorized into three types. Most kids showed yellow to yellow-green loose or watery diarrhea, often accompanied by fecal contamination around the perineum and hind limbs. Some kids presented with hemorrhagic diarrhea, characterized by red to dark red watery feces with a foul odor, frequently associated with acute onset, abnormal body temperature, weakness, and occasional neurological signs such as ataxia. The remaining cases mainly exhibited mucoid diarrhea, with grayish-yellow or green feces, occasionally containing small amounts of blood streaks.

### 3.2. Correlation Between Fecal Characteristics and Pathogen Infection

**Figure 7:**
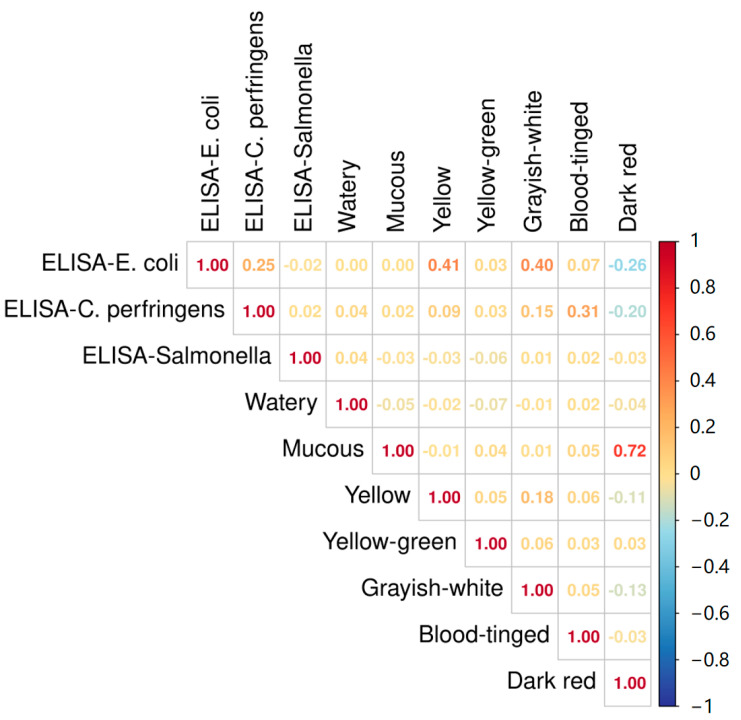
Pearson correlation analysis between clinical fecal characteristics and pathogen detection in dairy goat kids with diarrhea.

Pearson correlation analysis revealed that the positive rate of *Escherichia coli* was moderately positively correlated with yellow feces (r = 0.41) and gray-white feces (r = 0.40), and weakly correlated with the positive rate of *Clostridium perfringens* (r = 0.25). The positive rate of *C. perfringens* showed a weak positive correlation with bloody feces (r = 0.31). No significant correlations were observed between *Salmonella* spp. and fecal characteristics. Among fecal traits, mucoid feces were strongly positively correlated with dark red feces (r = 0.72) (Figure 7).

### 3.3. Distribution of Toxin Genotypes of Clostridium perfringens

A total of 73 *Clostridium perfringens* isolates were subjected to PCR detection of toxin genes. All isolates carried the *cpa* gene. Toxin genotyping (Table 6) showed that type B (*cpa*^+^*cpb*^+^*etx*^+^) was predominant (49/73, 67.1%), followed by type C (*cpa*^+^*cpb*^+^) (16/73, 21.9%), type A (*cpa*^+^) (5/73, 6.8%), and type D (*cpa*^+^*etx*^+^) (3/73, 4.1%). Type B isolates were distributed across all sampling regions, with the highest number in Helingeer (*n* = 19) (Table 6).

### 3.4. Antimicrobial Susceptibility of Clostridium perfringens

Antimicrobial susceptibility testing results (Table 7) showed that the majority of the 73 *Clostridium perfringens* isolates were highly susceptible to tetracycline, cefoxitin, penicillin, and metronidazole, with susceptibility rates exceeding 90%. Among these, tetracycline exhibited the highest susceptibility rate (94.5%).

In contrast, the highest resistance rate was observed for ampicillin (27.4%), followed by clindamycin (12.3%). Resistance to penicillin and metronidazole was relatively low, at 9.6% and 2.7%, respectively, while no resistance to cefoxitin and only minimal resistance to tetracycline (1.4%) were detected. The resistance rate to ampicillin was significantly higher than those to the other antibiotics (*p* < 0.05).

### 3.5. Comparative Metabolomic Analysis of Type B and Type C Clostridium perfringens

#### 3.5.1. Overview of Metabolomic Profiles and Multivariate Analysis

**Figure 8:**
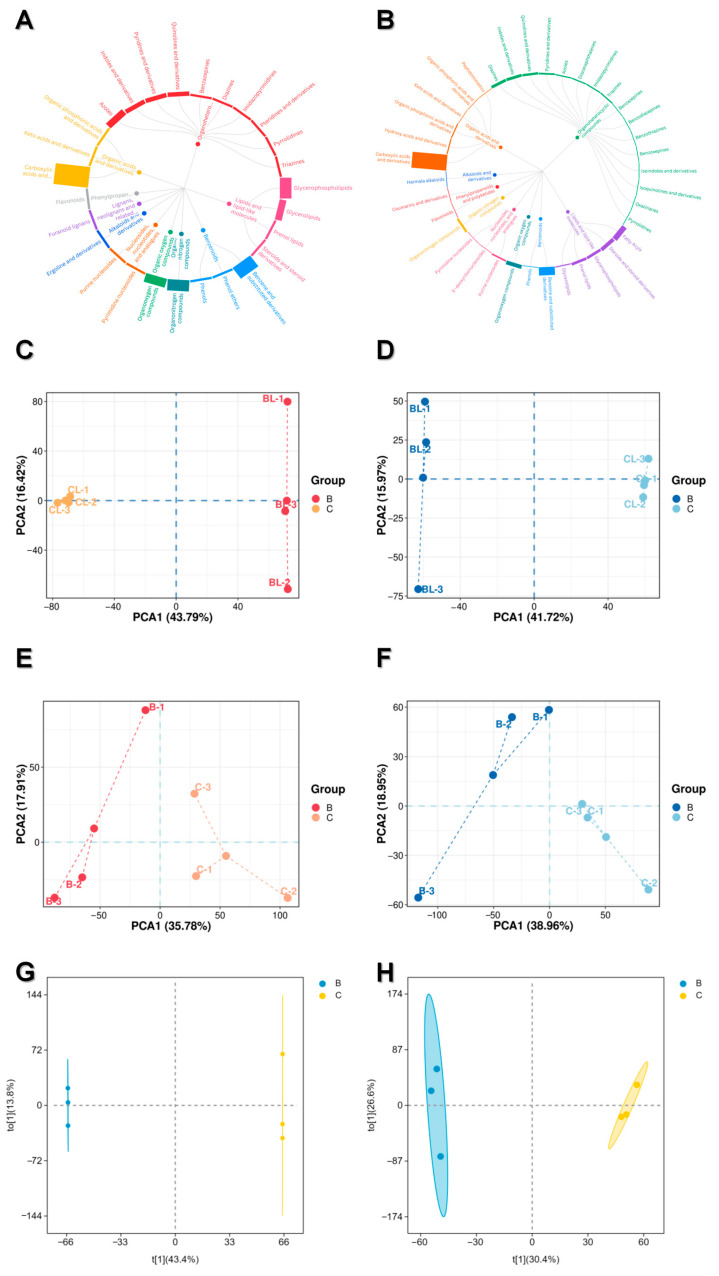
Integrated metabolomic analysis of type B and type C *Clostridium perfringens*. (**A**) Composition of differential metabolites in the culture supernatant; (**B**) composition of differential metabolites in the bacterial pellet. Principal component analysis (PCA) score plots of the culture supernatant under positive (**C**) and negative (**D**) ion modes. PCA score plots of the bacterial pellet under positive (**E**) and negative (**F**) ion modes. (**G**) OPLS-DA score plot of the culture supernatant; (**H**) OPLS-DA score plot of the bacterial pellet.

The composition of differential metabolites between type B and type C *Clostridium perfringens* is shown in Figure 8A,B. In both the culture supernatant and bacterial pellet, the differential metabolites were mainly classified into organic acids and derivatives, organic heterocyclic compounds, and lipids and lipid-like molecules. Principal component analysis (PCA) revealed a clear separation between type B and type C samples in both positive and negative ion modes for the culture supernatant (Figure 8C,D) and bacterial pellet (Figure 8E,F), with good clustering observed within each group. Furthermore, OPLS-DA analysis demonstrated a distinct discrimination between the two groups in both the culture supernatant and bacterial pellet (Figure 8G,H), indicating significant differences in metabolic profiles between type B and type C *C. perfringens*. These results collectively suggest substantial metabolic divergence between the two toxin types, with all differential metabolites showing statistical significance (*p* < 0.05).

#### 3.5.2. Identification of Differential Metabolites

**Figure 9:**
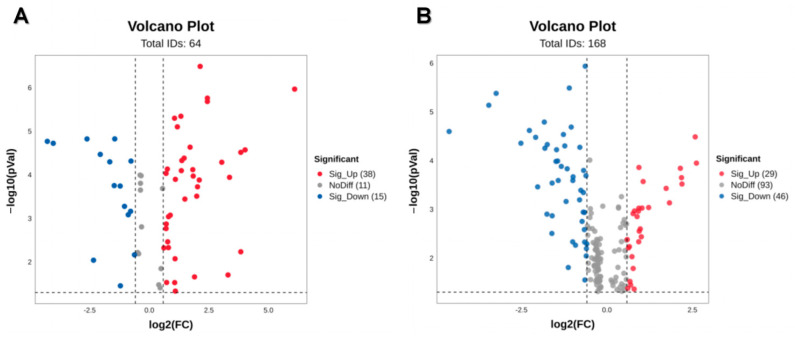
Volcano plots of differential metabolites between type B and type C *Clostridium perfringens*. (**A**) Culture supernatant; (**B**) Bacterial pellet. The dashed lines divide the heatmap into regions corresponding to upregulated, downregulated, and undetected metabolites, respectively.

Differential metabolites were screened based on VIP > 1, *p* < 0.05, and FC > 1.5 or <0.67. All identified differential metabolites met these criteria, confirming their statistical significance. A total of 53 significantly differential metabolites were identified in the culture supernatant, including 38 upregulated and 15 downregulated metabolites. In the bacterial pellet, 73 differential metabolites were identified, with 29 upregulated and 44 downregulated (Figure 9A,B).

## 4. Discussion

Diarrhea in neonatal kids is a major cause of mortality and reduced productivity in goat farming systems [1]. Previous studies have shown that the etiology of dairy kids’ diarrhea varies significantly across regions, highlighting the importance of identifying local dominant pathogens for targeted control strategies. In the present study, *Clostridium perfringens* was identified as the predominant pathogen in diarrheic kids, with a positive rate of 56.06%, followed by *Escherichia coli* (46.71%) and *Salmonella* spp. (24.54%). Mixed infections were also commonly observed. These findings are consistent with previous reports from Pakistan [13] and Egypt [14], where *C. perfringens* and *E. coli* were frequently identified as major pathogens in young ruminants, although detection rates varied across regions. Such differences may be attributed to environmental conditions, management practices, and detection methods. *C. perfringens* is an opportunistic pathogen widely distributed in soil, feed, and the gastrointestinal tract of healthy animals [15]. Under unfavorable conditions, such as poor hygiene or immune suppression, the bacterium can rapidly proliferate and produce potent toxins, leading to intestinal damage [16]. In this study, *C. perfringens* infection was positively correlated with bloody diarrhea, supporting its role in hemorrhagic enteritis. This is consistent with previous findings in calves and small ruminants [14,17]. The production of α, β, and ε toxins disrupts intestinal epithelial integrity and vascular function, resulting in increased intestinal permeability and severe enteric disease [15,18].

*E. coli*, another opportunistic pathogen, also showed a high detection rate and was positively correlated with yellow and gray-white feces. The weak positive correlation between *E. coli* and *C. perfringens* suggests the possibility of co-infection or secondary infection. It is likely that intestinal damage caused by *C. perfringens* facilitates the overgrowth of pathogenic *E. coli*, thereby exacerbating diarrheal symptoms [19].

*Clostridium perfringens* can be classified into seven toxinotypes (A–G), among which types A–D are most commonly associated with sheep and goats [6]. In the present study, type B was the predominant genotype (67.1%), followed by type C (21.9%), whereas types A and D were detected at much lower frequencies, indicating that type B is the dominant strain associated with dairy kids diarrhea in Inner Mongolia. Previous studies have demonstrated marked geographical variation in toxinotype distribution. For example, type B predominance has been reported in Iran [20], whereas type A was the dominant genotype in Saudi Arabia [21] and parts of China [22]. Collectively, these findings support the existence of strong regional differences in the epidemiology of *C. perfringens*.

Both type B and type C strains produce α- and β-toxins, while type B additionally produces ε-toxin, a highly potent pore-forming toxin. β-toxin is considered a key virulence factor responsible for vascular damage and hemorrhagic enteritis [23,24], whereas ε-toxin contributes to systemic toxicity and neurological symptoms. The clinical observations in the present study, including bloody diarrhea and occasional neurological signs, are consistent with the toxin profiles of type B strains [23,25].

In intensive goat production systems, antibiotic treatment is often applied empirically due to the rapid onset and progression of diarrheal diseases [26]. However, the widespread and sometimes excessive use of antibiotics has contributed to the emergence of antimicrobial resistance. In the present study, most *C. perfringens* isolates remained highly susceptible to tetracycline, cefoxitin, penicillin, and metronidazole, suggesting that these antibiotics may still be effective for clinical intervention. However, a relatively high resistance rate to ampicillin (27.4%) and moderate resistance to clindamycin (12.3%) were observed. Compared with previous studies from different regions and host species, variations in resistance profiles were evident, likely reflecting differences in antibiotic usage patterns [27]. These findings highlight the importance of continuous antimicrobial resistance surveillance and rational antibiotic use to minimize the development and spread of resistant strains.

Untargeted metabolomic analysis revealed significant differences between type B and type C *Clostridium perfringens* in both the culture supernatant and bacterial pellet. The culture supernatant mainly reflects extracellular metabolites such as secreted toxins and enzymes, whereas the bacterial pellet represents intracellular metabolic status and regulatory processes [28].

*C. perfringens* is an obligate anaerobe that generates ATP primarily through fermentation and substrate-level phosphorylation [9]. In this study, type B strains showed decreased levels of uridine, uracil, adenosine, and D-galactose, but increased hypoxanthine in both the culture supernatant and bacterial pellet. Uridine and uracil are key intermediates in pyrimidine metabolism and are essential for RNA synthesis, while adenosine is a precursor of ATP [29,30]. Their reduction in type B strains likely reflects rapid utilization to support active growth and energy production [30]. In contrast, the accumulation of hypoxanthine suggests enhanced ATP turnover and purine recycling. In addition, decreased levels of acetyl-CoA, D-glycerate, and L-fucose indicate accelerated carbon metabolism and increased flux through glycolysis and fermentation pathways [31,32].

Overall, these results suggest that type B strains possess a more efficient energy metabolism, which may support rapid proliferation and toxin synthesis.

Amino acid metabolism plays a critical role in nutrient acquisition and virulence [33]. In the culture supernatant, type B strains exhibited increased levels of proline-containing dipeptides, keto-leucine, and arginine. Proline-containing peptides are typically derived from host collagen degradation, suggesting enhanced proteolytic activity and tissue degradation capacity [34]. Keto-leucine, an intermediate of branched-chain amino acid metabolism, contributes to energy production and biosynthesis [34], while arginine can be utilized through the arginine deiminase pathway to generate ATP and support virulence factor synthesis [24].

In the bacterial pellet, increased levels of oligopeptides further indicate enhanced nutrient uptake and utilization capacity [35]. Meanwhile, decreased levels of metabolites such as citrulline and related compounds suggest increased metabolic flux through arginine metabolism and associated pathways [36,37]. These findings indicate that type B strains exhibit more active amino acid metabolism, which may provide both energy and biosynthetic substrates for rapid growth and toxin production.

Lipid metabolism in *Clostridium perfringens* is involved in maintaining cell membrane structure and mediates toxin activity as well as the activation of host cell signaling pathways through its metabolites [38,39,40]. In the present study, type B strains showed upregulation of various diacylglycerol (DAG) and phosphatidylethanolamine (PE) molecules in both the culture supernatant and bacterial pellet. These differential metabolites mainly included DAG and PE. DAG is an intermediate in fat storage and breakdown, primarily generated by phospholipase C (PLC)-mediated hydrolysis of phospholipids; it participates in cell signal transduction [41] and also serves as an intermediate in triglyceride synthesis [42]. Oda et al. [43] reported that the α-toxin of *C. perfringens* hydrolyzes phosphatidylcholine to produce DAG through its enzymatic activity, thereby activating host intracellular signaling pathways, which may be key to triggering severe symptoms such as gas gangrene. Takagishi et al. [44] found that DAG generation induced by α-toxin alters host cell membrane fluidity, promotes GM1a clustering and TrkA phosphorylation, and subsequently activates downstream inflammatory signaling pathways. The upregulation of DAG in type B strains may reflect its role as a signaling molecule involved in modulating host inflammation and energy storage. PE is one of the most abundant phospholipid components in bacterial cell membranes and plays an essential role in maintaining membrane fluidity, integrity, and function [45]. Increased PE levels in the bacterial pellet reflect rapid bacterial proliferation [46], while elevated PE in the culture supernatant may result from membrane components released upon cell lysis [47]. The upregulation of PE and related lipid molecules in type B strains suggests a stronger capacity for membrane lipid remodeling, supporting rapid proliferation and membrane structural stability. Their metabolite DAG may act as a signaling molecule that exacerbates intestinal inflammation in the host, which is consistent with the clinical manifestation of hemorrhagic enteritis caused by type B infection.

Virulence-related metabolism in *Clostridium perfringens* affects the maturation and activation of key virulence factors primarily by regulating post-translational processing of toxins and protease activity [48]. In the present study, type B strains exhibited downregulation of leupeptin and upregulation of fumagillin and bestatin compared with type C strains. Leupeptin is a broad-spectrum inhibitor of serine/cysteine proteases; it may affect toxin activation and delay toxin protein degradation by inhibiting proteases secreted by the bacterium itself. Shimizu et al. [48] reported, through proteomic analysis, that a clostripain-family cysteine protease exists among the VirR/VirS two-component regulatory virulence factors of *C. perfringens*, and that its activity is strongly inhibited by leupeptin, suggesting that leupeptin can act as an effective inhibitor of this key virulence-associated protease. Fumagillin inhibits methionine aminopeptidase 2, thereby removing the N-terminal methionine of nascent proteins [49,50]; bestatin inhibits aminopeptidases and is also involved in post-translational processing. The ε-toxin, uniquely produced by type B strains, is a secreted protein that depends on proper post-translational processing [49]. The upregulation of fumagillin and bestatin, together with the downregulation of leupeptin, suggests that type B strains may enhance the activity of the protein maturation pathway, providing a basis for efficient toxin production, ensuring rapid maturation and toxicity of the produced toxins, which explains the high pathogenicity characteristic of type B strains.

Several limitations of this study should be acknowledged. First, samples were collected from only four regions in Inner Mongolia, and the metabolomic analysis included only six isolates per toxinotype. Thus, caution is needed when generalizing our findings. Second, we focused only on bacterial pathogens; viral and parasitic causes of neonatal diarrhea of dairy goat kids were not examined. Third, our antimicrobial susceptibility results are from in vitro tests and may not fully reflect clinical efficacy; the resistance mechanism to ampicillin remains unclear. Finally, metabolomics revealed associations but not causal relationships between specific metabolites and the enhanced virulence of type B strains.

Future studies should cover more regions and include more isolates to confirm the predominance of type B. Integrated diagnostics for bacterial, viral, and parasitic agents are needed. Genomic approaches should be used to investigate antimicrobial resistance mechanisms. Targeted metabolomics and gene knockout experiments could clarify the metabolic basis of virulence. Field trials of alternative control strategies (e.g., probiotics, vaccination, improved management) are also warranted to reduce antibiotic use and disease impact in dairy goat farms.

## 5. Conclusions

This study identified *Clostridium perfringens* as the predominant pathogen associated with diarrhea in dairy goat kids in Inner Mongolia, with type B as the dominant genotype. The consistent predominance of type B across all sampling regions confirms its regional epidemiological significance. Most isolates remained susceptible to commonly used antibiotics, although resistance to ampicillin was relatively high, suggesting that ampicillin should be used with caution in clinical practice. Metabolomic analysis demonstrated that type B strains exhibit enhanced energy metabolism, amino acid utilization, lipid remodeling, and virulence-associated metabolic regulation compared with type C strains. These metabolic advantages may support rapid proliferation, toxin production, and increased pathogenicity, offering a metabolic perspective for understanding the predominance of this toxinotype. Overall, these findings provide important insights into the epidemiology and pathogenic mechanisms of *C. perfringens* in dairy goat kids and may contribute to the development of targeted prevention and control strategies, including regional vaccination programs, evidence-based antibiotic selection, and the exploration of metabolism-based intervention approaches.

## Figures and Tables

**Table 1:** Primers for toxin gene detection.

Gene	The Sequence of Primer (5′→3′)	Size (bp)
*Cpa* (α-toxin)	GCTAATGTTACTGCCGTTGA CCTCTGATACATCGTGTAAG	324
*Cpb* (β-toxin)	GCGAATATGCTGAATCATCTA GCAGGAACATTAGTATATCTTC	196
*Etx* (ε-toxin)	GCGGTGATATCCATCTATTC CCACTTACTTGTCCTACTAAC	665

**Table 2:** Interpretive criteria for antimicrobial susceptibility test.

Antibiotic Name	CLSI Interpretive Criteria (μg/mL)			Concentration Range (μg/mL)
	Susceptible (S)	Intermediate (I)	Resistant (R)	
Penicillin (PEN)	≤0.5	1	≥2	0.12–256
Metronidazole (MTZ)	≤8	16	≥32	0.12–256
Clindamycin (CLI)	≤2	4	≥8	0.06–128
Ampicillin (AMP)	≤0.5	1	≥2	0.12–256
Cefoxitin (FOX)	≤16	32	≥64	0.25–512
Tetracycline (TET)	≤4	8	≥16	0.12–256

**Table 3:** Biochemical identification record table for *Salmonella*.

Reaction Number	1	2	3	4	5	6	7	8	9	10	11	12	13
Reaction name	Mannitol	Sorbitol	Dulcitol	Salicin	ONPG	Malonate	Urea	Lysine	Amino acid control	H_2_S	Indole	KCL	Sodium chloride control
Result	+	+	+	-	-	-	-	+	Yellow	+	-	-	Turbid

**Table 4:** IMViC biochemical identification results of *Escherichia coli*.

Reaction Number	1	2	3	4
Reaction name	Indole (I)	Methyl red (M)	VP (V)	Citrate (C)
Result	+	+	-	-

**Table 5:** Serum antibody detection by ELISA in diarrheic and healthy dairy goat kids.

Pathogen	Diarrhea Group (*n* = 974)		Healthy Group (*n* = 157)	
	Positive Rate (%)	Positive (n/N)	Positive Rate (%)	Positive (n/N)
*Escherichia coli*	46.71	455/974	8.28	13/157
*Salmonella* spp.	24.54	239/974	3.82	6/157
*Clostridium perfringens*	56.06	546/974	1.27	2/157

**Table 6:** Distribution of *Clostridium perfringens* toxin genotypes by region.

Region	Type A	Type B	Type C	Type D
Helingeer	2	19	8	1
Liangcheng	0	12	2	1
Ordos	2	9	2	0
Bayannur	1	9	4	1
Total	5	49	16	3

**Table 7:** Antimicrobial susceptibility of *Clostridium perfringens* isolates (*n* = 73).

Antibiotic	Susceptible (n)	Intermediate (n)	Resistant (n)	Susceptible (%)	Resistant (%)
Penicillin (PEN)	66	0	7	90.41	9.59
Metronidazole (MTZ)	66	6	2	90.41	2.74
Clindamycin (CLI)	62	2	9	84.93	12.33
Ampicillin (AMP)	47	6	20	64.38	27.40
Cefoxitin (FOX)	67	6	0	91.78	0.00
Tetracycline (TET)	69	3	1	94.52	1.37

## Data Availability

The original contributions presented in the study are included in the Article Material; further inquiries can be directed to the corresponding author.
